# Dynamic perioperative thrombo-inflammatory biomarkers correlate with early functional outcomes after mechanical thrombectomy for acute ischemic stroke

**DOI:** 10.3389/fnins.2026.1922922

**Published:** 2026-10-07

**Authors:** Peihui Liu, Ying Chen, Di Shen, Tianyi Wang, Xue Han, Daguang Shen, He Sun, Jian Wang, Hongkui Wu, Tao Wang

**Affiliations:** Department of Neurointervention and Interventional Vascular Surgery, Huludao Central Hospital, Huludao, Liaoning, China

**Keywords:** acute ischaemic stroke, coagulation, D-dimer, functional outcome, mechanical thrombectomy, neutrophil-to-lymphocyte ratio, retrospective cohort, thrombo-inflammation

## Abstract

**Background:**

Inflammatory and coagulation responses continue to evolve after mechanical thrombectomy for acute ischaemic stroke. A single admission measurement may therefore miss clinically relevant perioperative biology. We examined whether longitudinal thrombo-inflammatory biomarker profiles were associated with early functional and neurological outcomes after thrombectomy.

**Methods:**

This single-centre retrospective cohort study grouped routine laboratory measurements into baseline (−3 to 0 days), postoperative days 1–3, 4–7, and 8–14. The primary endpoint was an unfavourable discharge modified Rankin Scale (mRS) score of 3–6. Failure of in-hospital early neurological improvement, defined as an admission-to-discharge NIHSS reduction of <4 points without a discharge NIHSS of 0–1, was examined as a secondary dynamic neurological endpoint. Biomarker associations were estimated using logistic regression adjusted for age, sex, and admission NIHSS. The clinical and combined models were compared in the same 586-patient complete-case subset.

**Results:**

Amongst 963 registry patients, 801 had discharge mRS data; 428 had favourable and 373 had unfavourable outcomes. Higher baseline NLR (OR 1.30, 95% CI 1.08–1.58), peak first-week NLR (OR 1.26, 95% CI 1.08–1.48), peak 1st-week D-dimer (OR 1.07, 95% CI 1.01–1.14), and lower baseline fibrinogen (OR 0.78, 95% CI 0.63–0.97) and minimum first-week lymphocyte count (OR 0.67, 95% CI 0.54–0.84) were independently associated with an unfavourable discharge outcome. Baseline platelet-to-lymphocyte ratio and white blood cell count on postoperative days 4–7 and 8–14 were also associated after adjustment. The principal biomarker signals were directionally consistent for failure of in-hospital early neurological improvement. In the matched subset, the clinical model had an AUC of 0.709 and the combined model an AUC of 0.726; the paired AUC difference was 0.017 (95% bootstrap CI −0.003 to 0.037).

**Conclusion:**

Dynamic perioperative thrombo-inflammatory profiles were associated with early disability and neurological recovery after thrombectomy. Their incremental discrimination beyond basic clinical variables was small and statistically uncertain. These findings are exploratory and require prospective, multicentre validation with standardised sampling before clinical implementation.

## 1 Introduction

Mechanical thrombectomy has transformed the treatment of acute ischaemic stroke caused by large-vessel occlusion. Recent randomised trials have extended the evidence base to selected patients with large infarct cores, broadening eligibility for endovascular therapy whilst also making post-procedural heterogeneity increasingly apparent (Huo et al., 2023; Sarraj et al., 2023; Bendszus et al., 2023; Costalat et al., 2024; Writing Committee for the TESLA Investigators and TESLA Investigators, 2024).

Angiographic reperfusion does not guarantee rapid neurological recovery. Age, baseline neurological severity, infarct burden, treatment delay, reperfusion quality, haemorrhagic transformation, tissue-level no-reflow, and malignant oedema may all influence outcome (Writing Committee for the TESLA Investigators and TESLA Investigators, 2024; Horie et al., 2025; Liu et al., 2026; Chen et al., 2025; Qiao et al., 2024; Pohlmann et al., 2024; Kim et al., 2024; Matsukawa et al., 2024; Sun et al., 2024). These clinical and imaging characteristics are important, but they do not fully describe the biological response that develops after recanalisation.

Thrombo-inflammatory biomarkers provide a practical window into this evolving response. Neutrophils may promote endothelial injury, blood–brain barrier disruption, and neutrophil extracellular trap formation; lymphopenia may reflect stress-related suppression of adaptive immunity; platelet activation links inflammation to microvascular thrombosis; and D-dimer and fibrinogen reflect fibrin turnover and coagulation activity. Accordingly, NLR, PLR, C-reactive protein-related indices, D-dimer, and fibrinogen have been associated with outcome after thrombectomy (Wang et al., 2024; Memis et al., 2024; Dogan et al., 2025; Prandin et al., 2025; Barakzie et al., 2025; Lin et al., 2024; Nam et al., 2023; Zhao et al., 2025; Jiang et al., 2025; Xu et al., 2024; Barakzie et al., 2025; Yilmaz and Sencan, 2025; Chen et al., 2024).

Most previous studies have evaluated biomarkers at a single time point, generally on admission or within the first postoperative day. This approach may miss clinically relevant changes driven by ischaemia-reperfusion injury, endothelial dysfunction, blood–brain barrier disruption, infarct evolution, infection, and early complications. A longitudinal profile may therefore provide information that is not captured by a single baseline value.

We investigated perioperative inflammatory and coagulation biomarker profiles in a real-world cohort of patients undergoing mechanical thrombectomy. We examined their associations with unfavourable discharge disability, assessed neurological change using serial NIHSS values, and quantified whether dynamic biomarkers added prognostic information beyond conventional clinical variables when both models were evaluated in the same patients.

## 2 Materials and methods

### 2.1 Study design and data source

This single-centre retrospective cohort study used an institutional mechanical thrombectomy registry linked to perioperative laboratory records. The registry contained demographic characteristics, vascular risk factors, admission stroke severity, imaging and procedural variables, and functional outcomes. Laboratory records were linked to the thrombectomy date to reconstruct perioperative inflammatory and coagulation profiles.

Laboratory testing followed routine clinical care rather than a study-mandated schedule. Consequently, the number and timing of measurements varied amongst patients and were influenced by clinical condition and length of hospitalisation.

### 2.2 Study population

Patients were eligible if they had acute ischaemic stroke, underwent mechanical thrombectomy, and had sufficient clinical information for outcome assessment. Patients without a discharge mRS score were excluded from the primary outcome analysis. Biomarker-specific analyses used available cases with a measurement in the relevant perioperative window; no unmeasured laboratory values were generated or imputed.

### 2.3 Clinical and procedural variables

Collected variables included age, sex, admission and discharge NIHSS scores, baseline Alberta Stroke Program Early CT Score (ASPECTS), medical history, Trial of Org 10,172 in Acute Stroke Treatment (TOAST) classification, successful reperfusion, and haemorrhagic transformation. Successful reperfusion was defined as a modified Thrombolysis in Cerebral Infarction score of 2b-3. Haemorrhagic transformation was identified from registry clinical and imaging records.

For an extended sensitivity model, available covariates additionally included baseline ASPECTS, successful reperfusion, haemorrhagic transformation, baseline creatinine, and baseline alanine aminotransferase. Infarct volume, collateral grade, exact onset-to-reperfusion time, peri-procedural blood-pressure trajectories, anaesthesia strategy, intravenous thrombolysis, and detailed antithrombotic exposure were not consistently available in the source registry and therefore could not be incorporated reliably.

### 2.4 Biomarker assessment and perioperative windows

The biomarker panel comprised white blood cell, neutrophil, lymphocyte, and platelet counts; NLR; PLR; C-reactive protein; D-dimer; fibrinogen; prothrombin time; activated partial thromboplastin time; and international normalised ratio. These markers were selected because they were available through routine hospital laboratory workflows and represented complementary inflammatory, cellular, platelet-related, and coagulation pathways.

Measurements were grouped into four clinically pragmatic windows: baseline (−3 to 0 days relative to thrombectomy), postoperative days 1–3, postoperative days 4–7, and postoperative days 8–14. When more than one value was available within a window, the median was used. For first-week summary analyses, peak values were calculated for NLR, D-dimer, fibrinogen, and C-reactive protein, whereas minimum values were calculated for lymphocyte and platelet counts.

### 2.5 Outcome definitions

The primary outcome was unfavourable early functional outcome, defined as a discharge mRS score of 3–6; scores of 0–2 were considered favourable. Discharge mRS was retained as the primary endpoint because it captures early disability and in-hospital complications, although it is sensitive to length of stay and local discharge practise.

To evaluate dynamic neurological change, we added a secondary in-hospital NIHSS endpoint amongst patients with numeric admission and discharge NIHSS scores. Early neurological improvement was defined as an NIHSS reduction of at least 4 points or a discharge NIHSS score of 0–1, consistent with commonly used early neurological improvement criteria (Lai et al., 2023). Failure to meet either criterion was treated as the adverse neurological endpoint. Because a uniform 24-h NIHSS assessment was not available retrospectively, this analysis represents admission-to-discharge neurological change rather than a standardised 24-h endpoint.

### 2.6 Missing data and risk of ascertainment bias

No statistical imputation was performed. Available-case denominators were reported for every biomarker and time window, and biomarker availability is summarised in Supplementary Table S1. Later postoperative measurements were conditional on continued hospitalisation and repeat testing; patients with greater severity, complications, or longer stays may therefore have been over-represented in the day 4–7 and day 8–14 windows. Late-window comparisons were interpreted as exploratory and potentially affected by informative missingness rather than as population-wide trajectories.

### 2.7 Statistical analysis

Continuous variables are reported as medians and interquartile ranges, and categorical variables as counts and percentages. Between-group comparisons used the Mann–Whitney U test for continuous variables and the chi-square or Fisher exact test for categorical variables, as appropriate.

Logistic regression was used to estimate associations with unfavourable discharge outcome. Biomarker odds ratios were scaled per interquartile-range increase. Primary adjusted models included age, sex, and admission NIHSS. Each biomarker was entered in a separate adjusted model to limit collinearity amongst biologically related markers.

To address potential selective reporting, regression analyses included not only the prespecified principal markers but also baseline neutrophil count, baseline PLR, and white blood cell count on postoperative days 4–7 and 8–14, which differed between outcome groups in descriptive analyses.

Four predictive models were evaluated: a clinical model (age, sex, and admission NIHSS), a baseline biomarker model (baseline white blood cell count, NLR, D-dimer, and fibrinogen), a dynamic biomarker model (peak first-week NLR, D-dimer, and fibrinogen and minimum first-week lymphocyte and platelet counts), and a combined clinical plus dynamic biomarker model. The primary comparison between the clinical and combined models was performed in the same 586-patient complete-case subset. AUC confidence intervals and the paired AUC difference were estimated by bootstrap resampling.

Sensitivity analyses examined 90-day mRS 3–6, discharge mRS 4–6, failure of in-hospital early neurological improvement, and an extended covariate model including ASPECTS, reperfusion, haemorrhagic transformation, creatinine, and alanine aminotransferase. False discovery rate correction was applied to selected repeated window comparisons.

All analyses were conducted in Python 3.12.13 using pandas, NumPy, SciPy, statsmodels, and scikit-learn. Tests were two-sided, and *p* < 0.05 was considered statistically significant unless otherwise stated.

## 3 Results

### 3.1 Study cohort and biomarker availability

The source registry contained 963 patients who underwent mechanical thrombectomy for acute ischaemic stroke. Discharge mRS was available for 801 patients, of whom 428 (53.4%) had a favourable outcome and 373 (46.6%) had an unfavourable outcome (Figures 1, 2).

**Figure 1:**
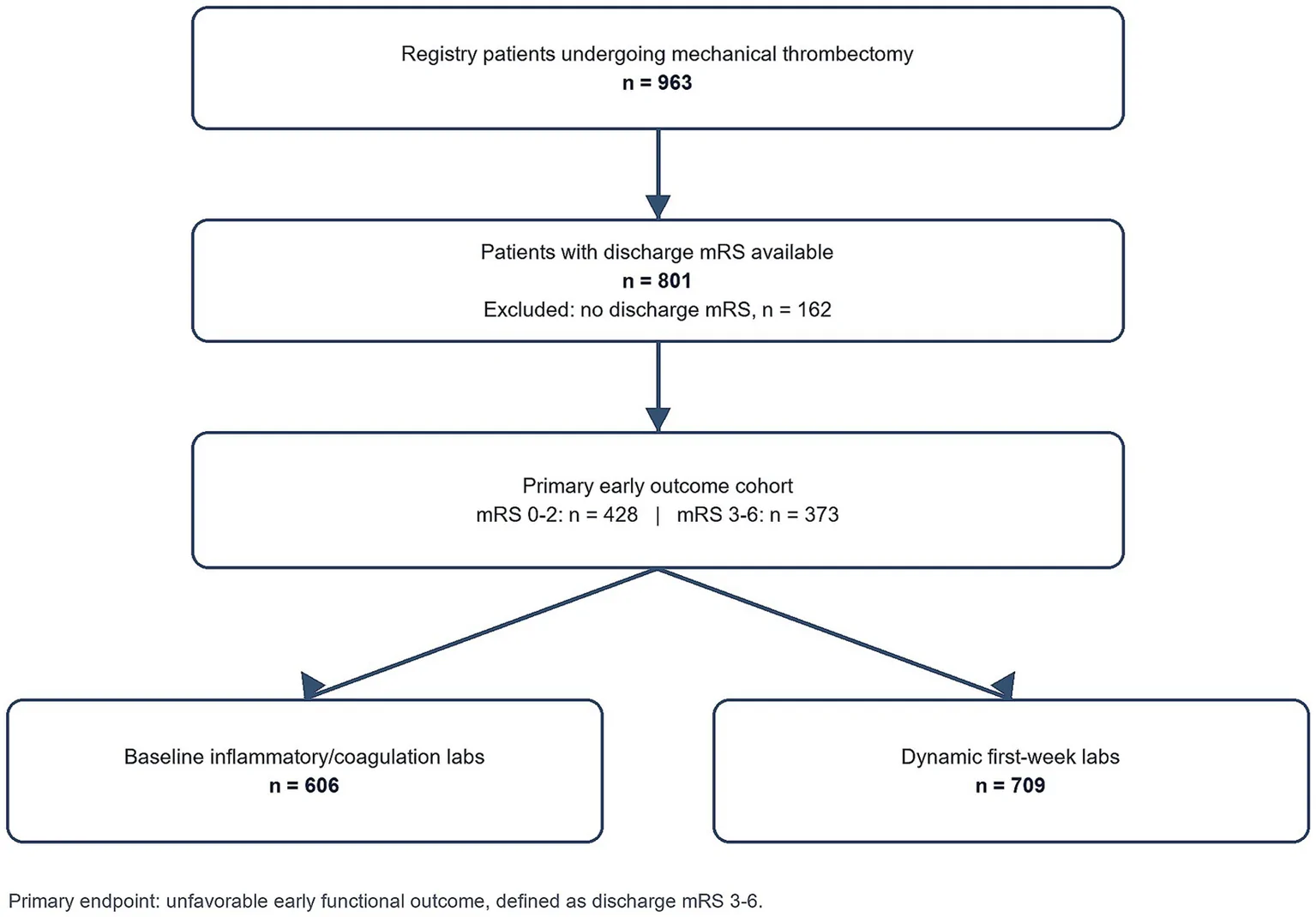
Cohort flow diagram. The source registry included 963 patients. Discharge mRS data were available for 801 patients, including 428 with favourable and 373 with unfavourable early functional outcomes. Baseline laboratory data were available for 606 patients and dynamic first-week data for 709 patients.

**Figure 2:**
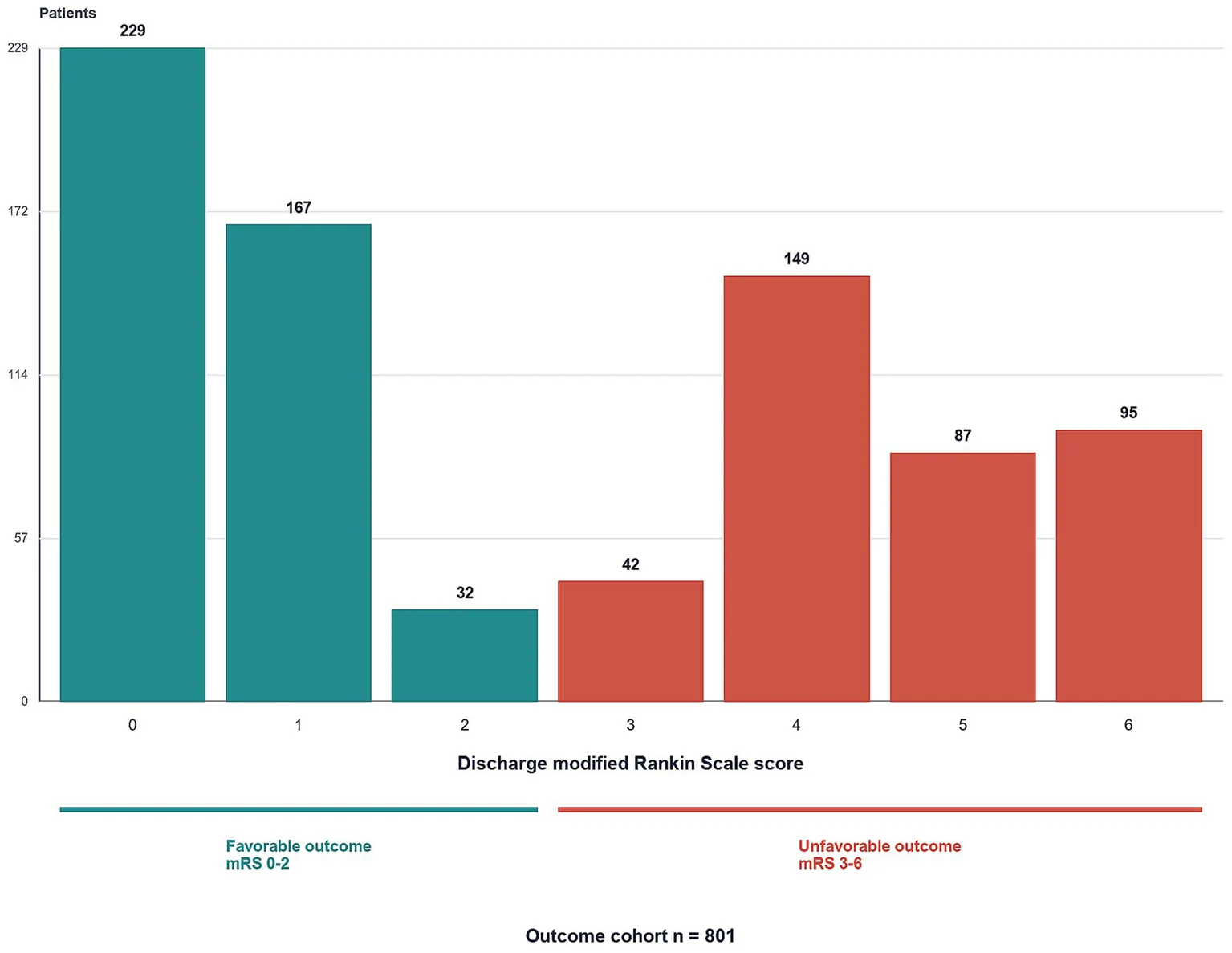
Distribution of discharge modified Rankin Scale scores. Bars show discharge mRS scores from 0 to 6. Scores of 0–2 were classified as favourable and scores of 3–6 as unfavourable early functional outcome.

Biomarker availability declined across later windows. For NLR, 514 patients had baseline data, 428 had postoperative day 1–3 data, 388 had day 4–7 data, and 281 had day 8–14 data. Baseline C-reactive protein was available in only 44 patients. Detailed availability for all markers is provided in Supplementary Table S1.

### 3.2 Clinical characteristics and neurological change

Patients with unfavourable outcomes were older and had more severe neurological deficits on admission (Table 1). Median admission NIHSS was 17 [11.5, 24.0] in the unfavourable group and 12 [7.0, 18.0] in the favourable group (*p* < 0.001). Successful reperfusion was less frequent (44.0% vs. 71.7%, *p* < 0.001), whereas haemorrhagic transformation was more frequent (29.2% vs. 17.8%, *p* < 0.001).

**Table 1:** Baseline clinical characteristics and in-hospital neurological change.

Characteristic	Overall	Favourable mRS 0–2	Unfavourable mRS 3–6	*p* value
Patients, *n*	801	428	373	
Age, years	66.0 [58.0, 72.0] (*n* = 801)	65.0 [57.0, 72.0] (*n* = 428)	67.0 [59.0, 74.0] (*n* = 373)	0.002
Male sex	578 (72.2) (*n* = 801)	317 (74.1) (*n* = 428)	261 (70.0) (*n* = 373)	0.207
Admission NIHSS	13.0 [9.0, 21.0] (*n* = 770)	12.0 [7.0, 18.0] (*n* = 411)	17.0 [11.5, 24.0] (*n* = 359)	<0.001
Discharge NIHSS	6.0 [1.0, 12.0] (*n* = 702)	2.0 [0.0, 4.0] (*n* = 425)	11.0 [8.0, 16.0] (*n* = 277)	<0.001
NIHSS improvement (admission minus discharge)	5.0 [1.0, 11.0] (*n* = 679)	8.0 [2.0, 13.0] (*n* = 409)	3.0 [0.0, 8.0] (*n* = 270)	<0.001
In-hospital early neurological improvement	443 (65.2) (*n* = 679)	315 (77.0) (*n* = 409)	128 (47.4) (*n* = 270)	<0.001
Baseline ASPECTS	8.0 [8.0, 9.0] (*n* = 801)	8.0 [8.0, 9.0] (*n* = 428)	8.0 [8.0, 9.0] (*n* = 373)	0.003
Successful reperfusion (mTICI 2b-3)	471 (58.8) (*n* = 801)	307 (71.7) (*n* = 428)	164 (44.0) (*n* = 373)	<0.001
Haemorrhagic transformation	185 (23.1) (*n* = 801)	76 (17.8) (*n* = 428)	109 (29.2) (*n* = 373)	<0.001
Atrial fibrillation history	160 (20.0) (*n* = 801)	83 (19.4) (*n* = 428)	77 (20.6) (*n* = 373)	0.659
Hypertension history	383 (47.8) (*n* = 801)	201 (47.0) (*n* = 428)	182 (48.8) (*n* = 373)	0.620
Diabetes history	170 (21.2) (*n* = 801)	86 (20.1) (*n* = 428)	84 (22.5) (*n* = 373)	0.436
Coronary disease history	62 (7.7) (*n* = 801)	26 (6.1) (*n* = 428)	36 (9.7) (*n* = 373)	0.064
TOAST: Other determined aetiology	4 (0.5)	2 (0.5)	2 (0.5)	0.381
TOAST: Large-artery atherosclerosis	429 (53.6)	240 (56.1)	189 (50.7)	
TOAST: Small-vessel occlusion	6 (0.7)	2 (0.5)	4 (1.1)	
TOAST: Cardioembolism	362 (45.2)	184 (43.0)	178 (47.7)	

Data are median [interquartile range] or *n* (%). Early neurological improvement was defined as an admission-to-discharge NIHSS reduction ≥4 points or discharge NIHSS 0–1. NIHSS, National Institutes of Health Stroke Scale; ASPECTS, Alberta Stroke Program Early CT Score; mTICI, modified Thrombolysis in Cerebral Infarction.

Amongst 679 patients with paired numeric NIHSS values, the median admission-to-discharge improvement was 5 [1, 11] points. In-hospital early neurological improvement occurred in 443 (65.2%) patients and was more frequent in the favourable mRS group than in the unfavourable group (77.0% vs. 47.4%, *p* < 0.001).

### 3.3 Baseline thrombo-inflammatory profiles

At baseline, the unfavourable outcome group had higher neutrophil count, higher NLR and PLR, and lower lymphocyte count (Table 2). Median NLR was 5.9 [3.6, 10.0] in the unfavourable group and 4.3 [3.0, 7.0] in the favourable group (*p* < 0.001). Baseline D-dimer did not differ significantly, whereas fibrinogen was modestly lower in the unfavourable group (2.6 [2.3, 3.1] vs. 2.7 [2.4, 3.2] g/L; *p* = 0.017).

**Table 2:** Baseline thrombo-inflammatory biomarker profiles.

Baseline biomarker	Overall	Favourable mRS 0–2	Unfavourable mRS 3–6	*p* value
White blood cell count, 10^9/L	8.6 [7.0, 10.7] (*n* = 514)	8.5 [7.0, 10.3] (*n* = 250)	8.9 [7.1, 10.9] (*n* = 264)	0.096
Neutrophil count, 10^9/L	6.6 [5.0, 8.7] (*n* = 514)	6.2 [4.8, 8.2] (*n* = 250)	7.0 [5.3, 9.1] (*n* = 264)	0.011
Lymphocyte count, 10^9/L	1.3 [0.9, 1.8] (*n* = 514)	1.4 [1.0, 1.8] (*n* = 250)	1.1 [0.8, 1.7] (*n* = 264)	<0.001
Platelet count, 10^9/L	205.0 [171.0, 248.4] (*n* = 514)	206.5 [173.5, 254.8] (*n* = 250)	203.5 [170.0, 239.0] (*n* = 264)	0.159
Neutrophil-to-lymphocyte ratio	5.0 [3.3, 8.6] (*n* = 514)	4.3 [3.0, 7.0] (*n* = 250)	5.9 [3.6, 10.0] (*n* = 264)	<0.001
Platelet-to-lymphocyte ratio	162.3 [116.1, 225.0] (*n* = 514)	149.2 [112.3, 204.2] (*n* = 250)	172.1 [120.5, 234.9] (*n* = 264)	0.010
C-reactive protein, mg/L	2.4 [1.0, 6.2] (*n* = 44)	2.0 [0.7, 5.7] (*n* = 18)	3.0 [1.3, 7.9] (*n* = 26)	0.257
D-dimer, mg/L	0.4 [0.2, 1.0] (*n* = 502)	0.4 [0.2, 1.0] (*n* = 245)	0.4 [0.2, 1.1] (*n* = 257)	0.271
Fibrinogen, g/L	2.7 [2.3, 3.2] (*n* = 505)	2.7 [2.4, 3.2] (*n* = 245)	2.6 [2.3, 3.1] (*n* = 260)	0.017
Prothrombin time, s	13.0 [12.4, 13.8] (*n* = 505)	12.9 [12.3, 13.7] (*n* = 245)	13.1 [12.4, 13.8] (*n* = 260)	0.300
Activated partial thromboplastin time, s	27.5 [24.8, 31.0] (*n* = 506)	27.8 [25.4, 30.9] (*n* = 246)	27.3 [24.3, 31.1] (*n* = 260)	0.069
International normalised ratio	1.1 [1.0, 1.2] (*n* = 505)	1.1 [1.0, 1.1] (*n* = 245)	1.1 [1.0, 1.2] (*n* = 260)	0.276

Data are median [interquartile range]. Denominators reflect available cases. NLR, neutrophil-to-lymphocyte ratio; PLR, platelet-to-lymphocyte ratio.

### 3.4 Dynamic perioperative trajectories

NLR was higher and lymphocyte count lower in the unfavourable group across all perioperative windows (Table 3; Figures 3, 4). D-dimer differed most clearly during postoperative days 1–3. C-reactive protein separated later, during postoperative days 4–7 and 8–14.

**Table 3:** Dynamic perioperative biomarker profiles.

Biomarker	Window	Overall	Favourable mRS 0–2	Unfavourable mRS 3–6	*p* value
Neutrophil-to-lymphocyte ratio	Baseline (−3 to 0 d)	5.0 [3.3, 8.6] (*n* = 514)	4.3 [3.0, 7.0] (*n* = 250)	5.9 [3.6, 10.0] (*n* = 264)	<0.001
Neutrophil-to-lymphocyte ratio	POD 1–3	6.6 [4.4, 11.3] (*n* = 428)	5.6 [3.9, 8.4] (*n* = 204)	7.9 [5.1, 13.1] (*n* = 224)	<0.001
Neutrophil-to-lymphocyte ratio	POD 4–7	4.6 [3.2, 7.4] (*n* = 388)	3.9 [2.9, 5.6] (*n* = 186)	5.4 [3.7, 8.3] (*n* = 202)	<0.001
Neutrophil-to-lymphocyte ratio	POD 8–14	4.1 [2.7, 6.3] (*n* = 281)	3.6 [2.5, 5.1] (*n* = 147)	4.7 [3.1, 9.2] (*n* = 134)	<0.001
White blood cell count, 10^9/L	Baseline (−3 to 0 d)	8.6 [7.0, 10.7] (*n* = 514)	8.5 [7.0, 10.3] (*n* = 250)	8.9 [7.1, 10.9] (*n* = 264)	0.096
White blood cell count, 10^9/L	POD 1–3	10.1 [8.1, 12.3] (*n* = 428)	9.6 [7.8, 11.7] (*n* = 204)	10.4 [8.3, 13.0] (*n* = 224)	0.011
White blood cell count, 10^9/L	POD 4–7	8.4 [6.8, 10.5] (*n* = 388)	7.8 [6.6, 10.0] (*n* = 186)	9.1 [7.5, 10.9] (*n* = 202)	0.001
White blood cell count, 10^9/L	POD 8–14	8.5 [7.0, 10.8] (*n* = 281)	8.3 [6.7, 9.7] (*n* = 147)	9.1 [7.2, 11.7] (*n* = 134)	0.008
Lymphocyte count, 10^9/L	Baseline (−3 to 0 d)	1.3 [0.9, 1.8] (*n* = 514)	1.4 [1.0, 1.8] (*n* = 250)	1.1 [0.8, 1.7] (*n* = 264)	<0.001
Lymphocyte count, 10^9/L	POD 1–3	1.2 [0.9, 1.6] (*n* = 428)	1.3 [1.1, 1.7] (*n* = 204)	1.1 [0.8, 1.5] (*n* = 224)	<0.001
Lymphocyte count, 10^9/L	POD 4–7	1.4 [1.0, 1.8] (*n* = 388)	1.4 [1.1, 1.9] (*n* = 186)	1.3 [0.9, 1.7] (*n* = 202)	0.002
Lymphocyte count, 10^9/L	POD 8–14	1.5 [1.1, 1.9] (*n* = 281)	1.6 [1.3, 1.9] (*n* = 147)	1.4 [1.1, 1.8] (*n* = 134)	0.004
Platelet count, 10^9/L	Baseline (−3 to 0 d)	205.0 [171.0, 248.4] (*n* = 514)	206.5 [173.5, 254.8] (*n* = 250)	203.5 [170.0, 239.0] (*n* = 264)	0.159
Platelet count, 10^9/L	POD 1–3	194.0 [159.8, 231.0] (*n* = 428)	197.0 [161.5, 235.1] (*n* = 204)	193.0 [157.8, 224.0] (*n* = 224)	0.223
Platelet count, 10^9/L	POD 4–7	203.0 [168.5, 248.2] (*n* = 388)	208.0 [174.0, 248.0] (*n* = 186)	194.5 [160.2, 248.4] (*n* = 202)	0.096
Platelet count, 10^9/L	POD 8–14	231.0 [187.0, 283.0] (*n* = 281)	232.0 [190.0, 282.5] (*n* = 147)	230.5 [181.0, 285.4] (*n* = 134)	0.444
D-dimer, mg/L	Baseline (−3 to 0 d)	0.4 [0.2, 1.0] (*n* = 502)	0.4 [0.2, 1.0] (*n* = 245)	0.4 [0.2, 1.1] (*n* = 257)	0.271
D-dimer, mg/L	POD 1–3	1.0 [0.4, 1.8] (*n* = 252)	0.8 [0.4, 1.7] (*n* = 115)	1.1 [0.5, 2.2] (*n* = 137)	0.016
D-dimer, mg/L	POD 4–7	1.1 [0.5, 2.8] (*n* = 202)	1.0 [0.4, 2.4] (*n* = 94)	1.3 [0.6, 3.5] (*n* = 108)	0.061
D-dimer, mg/L	POD 8–14	1.4 [0.6, 3.9] (*n* = 177)	1.3 [0.5, 3.3] (*n* = 93)	1.4 [0.7, 5.6] (*n* = 84)	0.176
Fibrinogen, g/L	Baseline (−3 to 0 d)	2.7 [2.3, 3.2] (*n* = 505)	2.7 [2.4, 3.2] (*n* = 245)	2.6 [2.3, 3.1] (*n* = 260)	0.017
Fibrinogen, g/L	POD 1–3	3.1 [2.6, 3.7] (*n* = 250)	3.2 [2.5, 3.9] (*n* = 114)	3.0 [2.6, 3.7] (*n* = 136)	0.964
Fibrinogen, g/L	POD 4–7	3.9 [3.2, 4.6] (*n* = 195)	3.8 [3.2, 4.5] (*n* = 91)	4.0 [3.3, 4.7] (*n* = 104)	0.194
Fibrinogen, g/L	POD 8–14	3.7 [2.9, 4.5] (*n* = 174)	3.7 [3.0, 4.6] (*n* = 89)	3.7 [2.9, 4.5] (*n* = 85)	0.984
C-reactive protein, mg/L	Baseline (−3 to 0 d)	2.4 [1.0, 6.2] (*n* = 44)	2.0 [0.7, 5.7] (*n* = 18)	3.0 [1.3, 7.9] (*n* = 26)	0.257
C-reactive protein, mg/L	POD 1–3	24.5 [8.2, 61.8] (*n* = 124)	27.5 [6.6, 48.0] (*n* = 52)	23.2 [10.1, 68.9] (*n* = 72)	0.611
C-reactive protein, mg/L	POD 4–7	30.7 [9.9, 66.8] (*n* = 113)	16.9 [7.0, 47.0] (*n* = 53)	44.7 [16.3, 80.2] (*n* = 60)	0.016
C-reactive protein, mg/L	POD 8–14	20.6 [8.1, 52.7] (*n* = 99)	12.9 [7.8, 38.1] (*n* = 45)	28.8 [11.2, 57.6] (*n* = 54)	0.025
Prothrombin time, s	Baseline (−3 to 0 d)	13.0 [12.4, 13.8] (*n* = 505)	12.9 [12.3, 13.7] (*n* = 245)	13.1 [12.4, 13.8] (*n* = 260)	0.300
Prothrombin time, s	POD 1–3	13.2 [12.5, 14.1] (*n* = 251)	13.2 [12.5, 14.0] (*n* = 115)	13.3 [12.5, 14.2] (*n* = 136)	0.974
Prothrombin time, s	POD 4–7	13.6 [12.8, 14.5] (*n* = 198)	13.5 [12.8, 14.4] (*n* = 93)	13.7 [12.8, 14.6] (*n* = 105)	0.692
Prothrombin time, s	POD 8–14	13.9 [12.9, 14.8] (*n* = 178)	13.6 [12.9, 14.6] (*n* = 92)	14.0 [12.9, 15.3] (*n* = 86)	0.086
Activated partial thromboplastin time, s	Baseline (−3 to 0 d)	27.5 [24.8, 31.0] (*n* = 506)	27.8 [25.4, 30.9] (*n* = 246)	27.3 [24.3, 31.1] (*n* = 260)	0.069
Activated partial thromboplastin time, s	POD 1–3	27.8 [24.9, 30.8] (*n* = 250)	27.4 [25.0, 29.8] (*n* = 114)	28.2 [24.6, 31.1] (*n* = 136)	0.771
Activated partial thromboplastin time, s	POD 4–7	28.7 [25.6, 32.8] (*n* = 195)	28.0 [25.6, 31.9] (*n* = 91)	29.2 [25.7, 33.6] (*n* = 104)	0.280
Activated partial thromboplastin time, s	POD 8–14	28.9 [26.5, 31.8] (*n* = 174)	29.4 [26.7, 31.9] (*n* = 89)	28.7 [26.1, 31.3] (*n* = 85)	0.453

Values are medians [interquartile range]. POD, postoperative day. Denominators vary by marker and window because testing followed routine clinical care.

**Figure 3:**
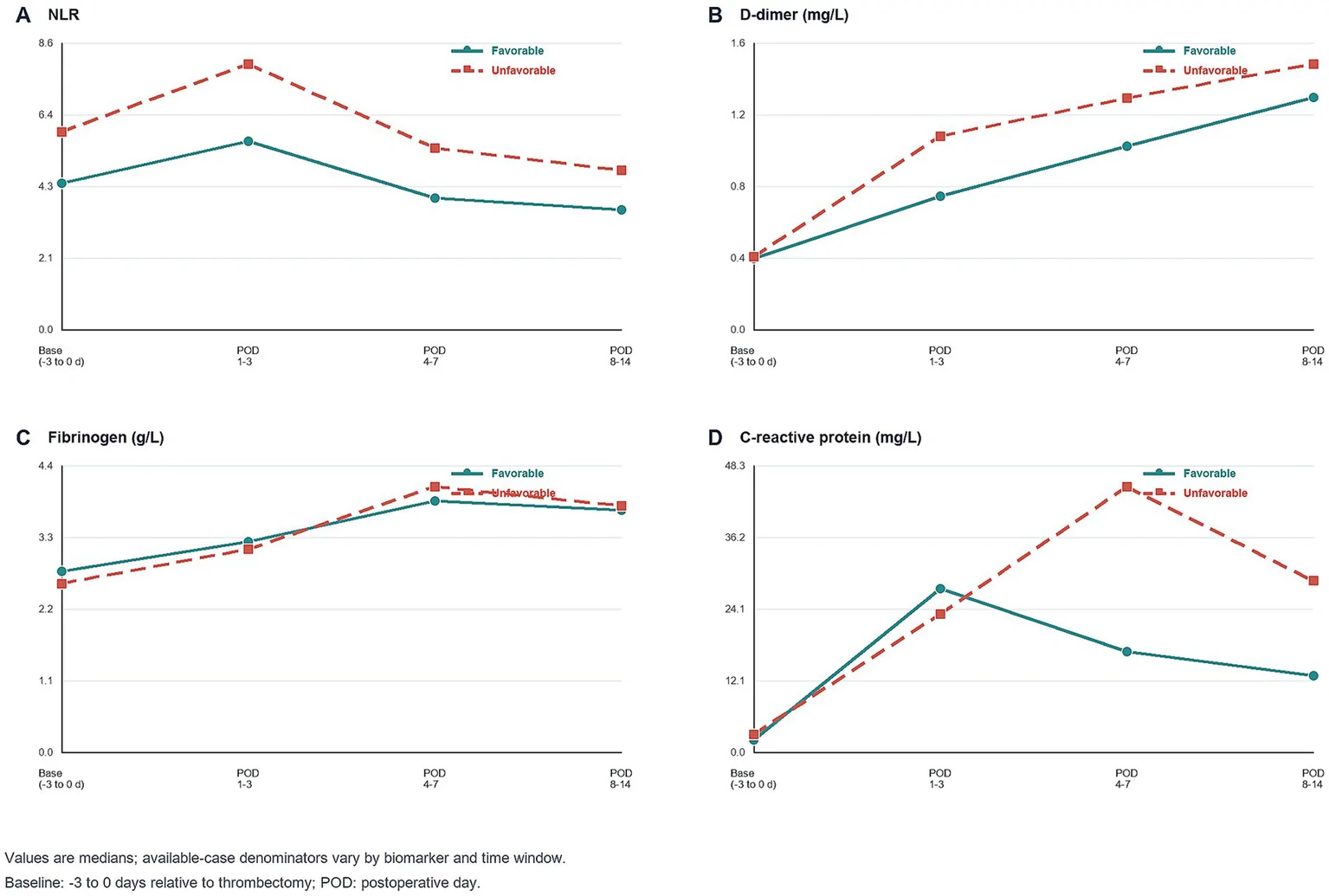
Dynamic perioperative biomarker trajectories according to early functional outcome. Points represent medians within each window; no error bars are shown. Available-case denominators vary by biomarker and time window and are reported in Table 3 and Supplementary Table S1.

**Figure 4:**
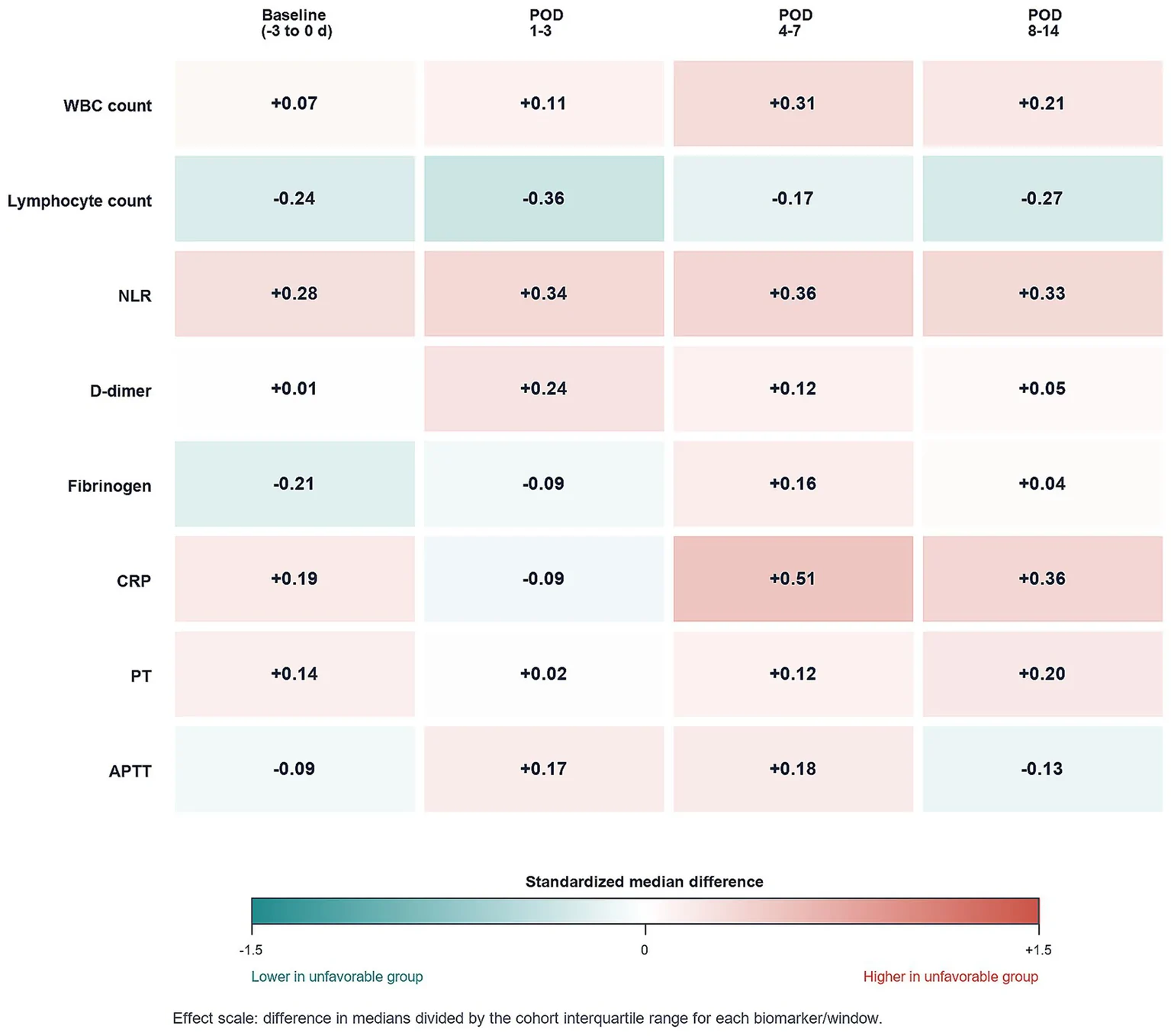
Standardised median-difference heatmap for perioperative biomarkers. Positive values indicate higher values in patients with an unfavourable outcome, and negative values indicate lower values. The heatmap is descriptive and should be interpreted in light of window-specific data availability.

White blood cell count was also higher in the unfavourable group on postoperative days 1–3, 4–7, and 8–14. Because later measurements were available predominantly in patients who remained hospitalised and underwent repeated testing, these late-window differences may reflect both biological progression and severity-related ascertainment.

### 3.5 Clinical and biomarker associations with unfavourable outcome

In univariable analyses, older age, higher admission NIHSS, lower ASPECTS, unsuccessful reperfusion, haemorrhagic transformation, higher baseline and peak first-week NLR, higher peak first-week D-dimer, lower baseline fibrinogen, and lower minimum first-week lymphocyte count were associated with unfavourable discharge outcome (Table 4).

**Table 4:** Univariable logistic regression for unfavourable discharge outcome.

Variable	Scale	*N*	Events	OR (95% CI)	*p* value
Age	Per 10-year increase	801	373	1.22 (1.08–1.39)	0.002
Male sex	Yes vs. No	801	373	0.82 (0.60–1.11)	0.198
Admission NIHSS	Per 5-point increase	770	359	1.41 (1.29–1.54)	<0.001
Baseline ASPECTS	Per 1-point increase	801	373	0.82 (0.72–0.94)	0.004
Successful reperfusion	Yes vs. No	801	373	0.31 (0.23–0.41)	<0.001
Hemorrhagic transformation	Yes vs. No	801	373	1.91 (1.37–2.67)	<0.001
Baseline neutrophil count	Per IQR increase	514	264	1.19 (0.98–1.46)	0.083
Baseline platelet-to-lymphocyte ratio	Per IQR increase	514	264	1.36 (1.11–1.65)	0.002
White blood cell count, POD 4–7	Per IQR increase	388	202	1.51 (1.16–1.97)	0.002
White blood cell count, POD 8–14	Per IQR increase	281	134	1.53 (1.17–2.01)	0.002
Baseline NLR	Per IQR increase	514	264	1.37 (1.13–1.65)	0.001
Peak NLR, days 0–7	Per IQR increase	612	312	1.35 (1.15–1.58)	<0.001
Baseline D-dimer	Per IQR increase	502	257	1.04 (0.99–1.09)	0.126
Peak D-dimer, days 0–7	Per IQR increase	587	301	1.09 (1.02–1.16)	0.007
Baseline fibrinogen	Per IQR increase	505	260	0.80 (0.65–0.98)	0.029
Peak fibrinogen, days 0–7	Per IQR increase	588	301	1.09 (0.91–1.30)	0.365
Peak CRP, days 0–7	Per IQR increase	204	111	1.17 (0.92–1.48)	0.212
Minimum lymphocytes, days 0–7	Per IQR increase	612	312	0.62 (0.50–0.76)	<0.001
Minimum platelets, days 0–7	Per IQR increase	612	312	0.84 (0.70–1.02)	0.075

Biomarker effects are scaled per interquartile-range increase. OR, odds ratio; CI, confidence interval.

The additional markers requested during review were analysed explicitly. Baseline neutrophil count was not independently associated after adjustment (OR 1.18, 95% CI 0.96–1.46; *p* = 0.121), whereas baseline PLR (OR 1.31, 95% CI 1.07–1.61; *p* = 0.010), white blood cell count on postoperative days 4–7 (OR 1.61, 95% CI 1.20–2.16; *p* = 0.001), and white blood cell count on postoperative days 8–14 (OR 1.50, 95% CI 1.13–2.00; *p* = 0.006) remained associated with unfavourable outcome (Table 5; Figure 5).

**Table 5:** Adjusted biomarker associations with unfavourable discharge outcome.

Variable	Scale	*N*	Events	Adjusted OR (95% CI)	*p* value
Baseline neutrophil count	Per IQR increase	514	264	1.18 (0.96–1.46)	0.121
Baseline platelet-to-lymphocyte ratio	Per IQR increase	514	264	1.31 (1.07–1.61)	0.010
White blood cell count, POD 4–7	Per IQR increase	388	202	1.61 (1.20–2.16)	0.001
White blood cell count, POD 8–14	Per IQR increase	281	134	1.50 (1.13–2.00)	0.006
Baseline NLR	Per IQR increase	514	264	1.30 (1.08–1.58)	0.007
Peak NLR, days 0–7	Per IQR increase	612	312	1.26 (1.08–1.48)	0.004
Baseline D-dimer	Per IQR increase	502	257	1.03 (0.98–1.08)	0.277
Peak D-dimer, days 0–7	Per IQR increase	587	301	1.07 (1.01–1.14)	0.026
Baseline fibrinogen	Per IQR increase	505	260	0.78 (0.63–0.97)	0.027
Peak fibrinogen, days 0–7	Per IQR increase	588	301	1.08 (0.89–1.30)	0.460
Peak CRP, days 0–7	Per IQR increase	204	111	1.16 (0.89–1.50)	0.272
Minimum lymphocytes, days 0–7	Per IQR increase	612	312	0.67 (0.54–0.84)	<0.001
Minimum platelets, days 0–7	Per IQR increase	612	312	0.88 (0.72–1.08)	0.234

Each biomarker was analysed in a separate model adjusted for age, sex, and admission NIHSS. Effects are scaled per interquartile-range increase.

**Figure 5:**
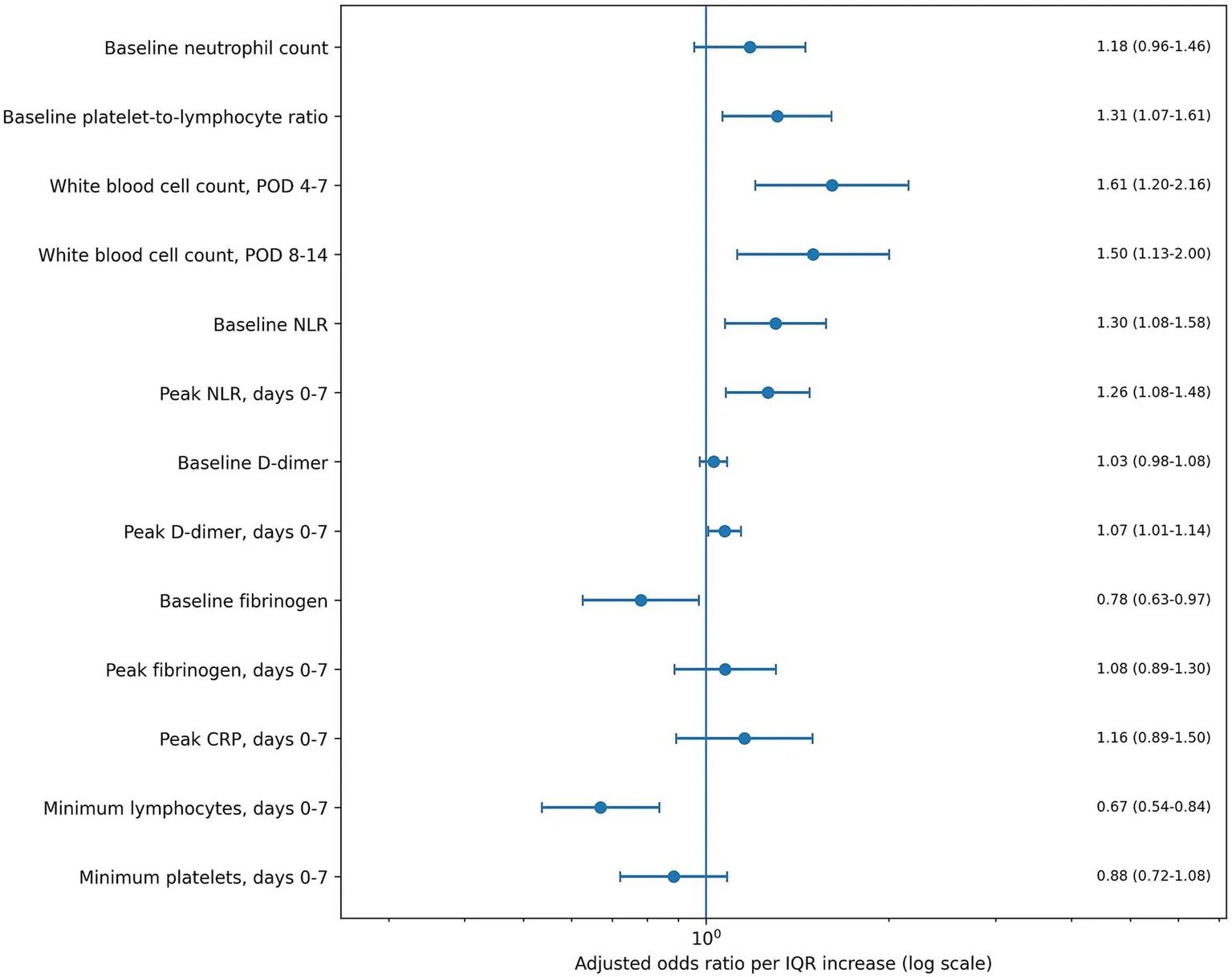
Adjusted odds ratios for unfavourable early functional outcome. Odds ratios and 95% confidence intervals are shown for prespecified and reviewer-requested biomarkers. Effects are scaled per interquartile-range increase and adjusted separately for age, sex, and admission NIHSS.

The principal adjusted associations were higher baseline NLR (OR 1.30, 95% CI 1.08–1.58; *p* = 0.007), higher peak first-week NLR (OR 1.26, 95% CI 1.08–1.48; *p* = 0.004), higher peak first-week D-dimer (OR 1.07, 95% CI 1.01–1.14; *p* = 0.026), lower baseline fibrinogen (OR 0.78, 95% CI 0.63–0.97; *p* = 0.027), and lower minimum first-week lymphocyte count (OR 0.67, 95% CI 0.54–0.84; *p* < 0.001).

### 3.6 Discriminative performance

In the full clinical cohort, the clinical model had an AUC of 0.668 (95% CI 0.630–0.706). In the 586 patients with complete dynamic biomarker data, the refitted clinical model had an AUC of 0.709 (95% CI 0.665–0.755), whilst the combined clinical plus dynamic biomarker model had an AUC of 0.726 (95% CI 0.682–0.766) (Table 6; Figure 6). The paired AUC increase was 0.017 (95% bootstrap CI −0.003 to 0.037), indicating only a small numerical increment without conclusive evidence of improved discrimination.

**Table 6:** Discriminative performance of clinical and biomarker models.

Model	*N*	Events	AUC (95% CI)
Clinical model, full clinical cohort	770	359	0.668 (0.630–0.706)
Clinical model, matched dynamic-biomarker subset	586	301	0.709 (0.665–0.755)
Baseline biomarker model	495	256	0.621 (0.573–0.669)
Dynamic biomarker model	586	301	0.631 (0.587–0.674)
Combined clinical + dynamic biomarkers	586	301	0.726 (0.682–0.766)

The primary comparison used the same 586-patient subset. The paired AUC difference between the combined and matched clinical models was 0.017 (95% bootstrap CI −0.003 to 0.037). AUC, area under the receiver operating characteristic curve.

**Figure 6:**
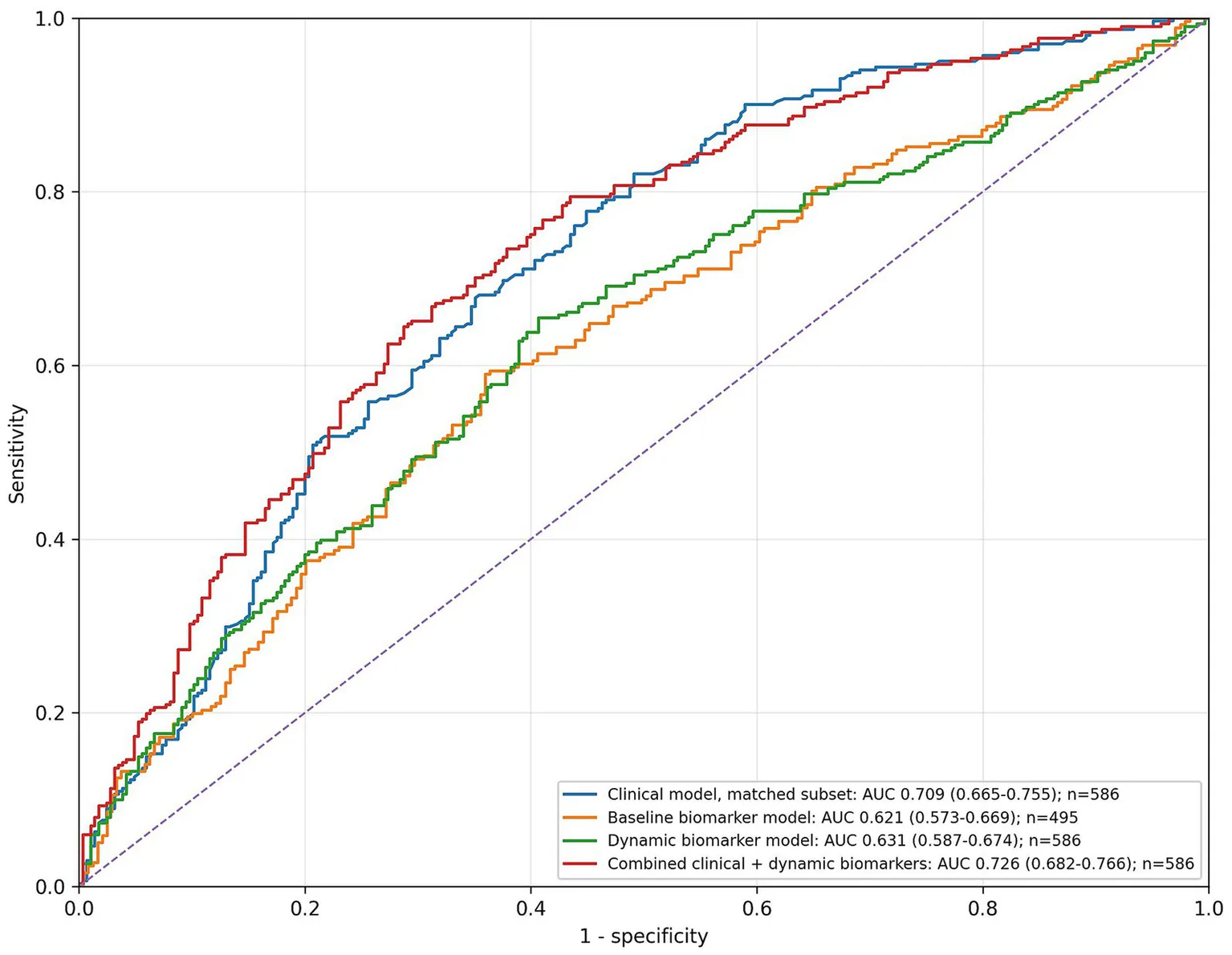
Receiver operating characteristic curves in the matched dynamic-biomarker subset (*n* = 586). The clinical model and combined clinical plus dynamic biomarker model were evaluated in the same patients. The AUC increased from 0.709 to 0.726; the paired difference was 0.017 (95% bootstrap CI −0.003 to 0.037).

### 3.7 Sensitivity analyses

The main biomarker signals were directionally similar when unfavourable outcome was defined as 90-day mRS 3–6 or discharge mRS 4–6.

For failure of in-hospital early neurological improvement, adjusted associations were observed for baseline NLR (OR 1.23, 95% CI 1.04–1.44), peak first-week NLR (OR 1.20, 95% CI 1.04–1.38), peak first-week D-dimer (OR 1.09, 95% CI 1.03–1.16), baseline fibrinogen (OR 0.74, 95% CI 0.58–0.96), and minimum first-week lymphocyte count (OR 0.65, 95% CI 0.50–0.85) (Supplementary Table S2).

In the extended available-case model including ASPECTS, reperfusion, haemorrhagic transformation, creatinine, and alanine aminotransferase, several estimates were attenuated. Peak first-week NLR and white blood cell count on postoperative days 8–14 remained statistically significant, whereas the confidence intervals for baseline NLR, baseline PLR, peak D-dimer, baseline fibrinogen, minimum lymphocyte count, and white blood cell count on days 4–7 included the null (Supplementary Table S3).

## 4 Discussion

### 4.1 Principal findings

This study yielded four main findings. First, an unfavourable discharge outcome was characterised by persistently higher NLR and lower lymphocyte counts across perioperative windows. Second, peak first-week D-dimer and lower baseline fibrinogen provided complementary coagulation signals. Third, baseline PLR and later white blood cell counts were associated with outcome after adjustment, although late-window results are vulnerable to informative testing and length-of-stay bias. Fourth, after a fair comparison in the same 586 patients, adding dynamic biomarkers increased the AUC by only 0.017 and the bootstrap confidence interval crossed zero. Thus, the biomarker profile was associated with outcome but did not establish a clinically validated prediction tool.

### 4.2 Inflammatory imbalance after thrombectomy

Persistent NLR elevation may reflect sustained innate immune activation together with suppression of adaptive immune responses. Activated neutrophils can promote endothelial injury, blood–brain barrier disruption, oxidative stress, and neutrophil extracellular trap formation, whilst lymphopenia may indicate stress-mediated immunosuppression and vulnerability to infection. The inverse association of minimum first-week lymphocyte count with adverse outcome suggests that the NLR signal was not driven solely by neutrophilia (Gao et al., 2024; Cao et al., 2024; Shi et al., 2025; Delibaş Kati and Uçan Tokuç, 2025; Prandin et al., 2025; Long et al., 2026; Zheng et al., 2024; Wang et al., 2024; Prandin et al., 2025; Barakzie et al., 2025; Lin et al., 2024).

PLR adds a platelet-related dimension to this interpretation. Platelets participate in leukocyte recruitment, endothelial adhesion, and thrombo-inflammatory microvascular obstruction; a higher PLR may therefore integrate platelet activity with relative lymphopenia. By contrast, late white blood cell elevations are nonspecific and may reflect infarct progression, infection, tissue injury, or other complications. Their apparent prognostic value should be interpreted cautiously because repeat measurements were more likely in patients with prolonged or complicated hospital courses.

### 4.3 Coagulation markers and early outcome

Baseline D-dimer did not clearly separate outcome groups, whereas peak first-week D-dimer was associated with unfavourable outcome. This time-dependent pattern is biologically plausible because D-dimer reflects ongoing fibrin formation and degradation and may increase with recurrent thrombosis, reperfusion injury, venous thromboembolism, or systemic complications (Nam et al., 2023; Zhang et al., 2024; Zhao et al., 2025; Jiang et al., 2025; Xu et al., 2024; Barakzie et al., 2025).

The inverse association between baseline fibrinogen and adverse outcome is more difficult to interpret. Fibrinogen is both a coagulation factor and an acute-phase reactant, and its association with stroke prognosis may be nonlinear and influenced by consumption, hepatic synthesis, timing, infarct severity, and systemic illness (Yilmaz and Sencan, 2025; Chen et al., 2024). This finding should therefore be considered hypothesis-generating rather than mechanistic proof.

### 4.4 Clinical and research implications

The studied biomarkers are inexpensive and routinely available, making longitudinal assessment feasible in many stroke centres. However, biomarker-only models showed limited discrimination and the incremental AUC in the paired comparison was small. These measurements may offer contextual information for post-thrombectomy monitoring, but they should not be used as stand-alone bedside decision thresholds.

Prospective studies should prespecify sampling times relative to reperfusion, standardise neurological assessments, and capture infection, infarct volume, collateral status, blood-pressure exposure, thrombolysis, antithrombotic treatment, and organ function. Broader panels incorporating markers of astroglial injury, neuronal damage, thrombin generation, endothelial dysfunction, and cytokine activation—including GFAP, NSE, thrombin-antithrombin complexes, thrombomodulin, and interleukin-6—may clarify whether dynamic profiles identify modifiable biological pathways rather than simply reflecting injury severity.

### 4.5 Strengths and limitations

Strengths include a relatively large real-world thrombectomy cohort, linkage of clinical and serial laboratory data, evaluation of both inflammatory and coagulation pathways, and sensitivity analyses using alternative outcomes and covariate sets.

Several limitations require emphasis. First, the retrospective single-centre design limits causal inference and generalisability. Laboratory measurements were obtained during routine care and were not missing at random. Patients who were clinically stable may have undergone fewer tests, whereas patients with greater severity, complications, or longer hospital stays were more likely to contribute data to later windows. This creates marker- and window-specific selection bias beyond a generic missing-data limitation.

Second, discharge mRS may be influenced by length of stay, discharge protocols, access to rehabilitation, and local practise. The added NIHSS analysis captured admission-to-discharge neurological change and supported the principal associations, but it did not replace a standardised 24-h NIHSS endpoint. Third, important covariates—including infarct volume, collateral circulation, exact onset-to-reperfusion time, peri-procedural blood-pressure management, anaesthesia, intravenous thrombolysis, and detailed antithrombotic treatment—were unavailable or incomplete, and residual confounding remains likely.

Fourth, pragmatic windows and summary values may smooth short-lived peaks or nadirs and do not capture the full trajectory shape. Finally, all models were exploratory and internally assessed. No external validation or clinical-utility study was performed; the reported AUCs must not be interpreted as evidence that the model is ready for bedside decision-making.

## 5 Conclusion

Dynamic perioperative thrombo-inflammatory biomarkers were associated with early functional disability and in-hospital neurological recovery after mechanical thrombectomy. Persistent NLR elevation, lymphopenia, higher early postoperative D-dimer, lower baseline fibrinogen, higher baseline PLR, and later white blood cell elevation were the principal signals. When evaluated in the same patients, adding dynamic biomarkers to basic clinical variables produced only a small and statistically uncertain improvement in discrimination. Prospective multicentre validation with standardised biomarker sampling and neurological follow-up is required before clinical use.

## Data Availability

The raw data supporting the conclusions of this article will be made available by the authors, without undue reservation.
