# Supplementary material for: Dynamic perioperative thrombo-inflammatory biomarkers correlate with early functional outcomes after mechanical thrombectomy for acute ischemic stroke
Source: Front Neurosci. 2026 Oct 7;20:1922922. doi: 10.3389/fnins.2026.1922922 (PMC13684488; doi:10.3389/fnins.2026.1922922)
Supplement: Supplementary file 1 [file Supplementary_file_1.DOCX]

**Supplementary Table S1. Availability of biomarker measurements by perioperative window.**

| **Biomarker** | **Baseline (-3 to 0 d)** | **POD 1-3** | **POD 4-7** | **POD 8-14** |
| --- | --- | --- | --- | --- |
| White blood cell count | 514 (64.2%) | 428 (53.4%) | 388 (48.4%) | 281 (35.1%) |
| Neutrophil count | 514 (64.2%) | 428 (53.4%) | 388 (48.4%) | 281 (35.1%) |
| Lymphocyte count | 514 (64.2%) | 428 (53.4%) | 388 (48.4%) | 281 (35.1%) |
| Platelet count | 514 (64.2%) | 428 (53.4%) | 388 (48.4%) | 281 (35.1%) |
| Neutrophil-to-lymphocyte ratio | 514 (64.2%) | 428 (53.4%) | 388 (48.4%) | 281 (35.1%) |
| Platelet-to-lymphocyte ratio | 514 (64.2%) | 428 (53.4%) | 388 (48.4%) | 281 (35.1%) |
| C-reactive protein | 44 (5.5%) | 124 (15.5%) | 113 (14.1%) | 99 (12.4%) |
| D-dimer | 502 (62.7%) | 252 (31.5%) | 202 (25.2%) | 177 (22.1%) |
| Fibrinogen | 505 (63.0%) | 250 (31.2%) | 195 (24.3%) | 174 (21.7%) |
| Prothrombin time | 505 (63.0%) | 251 (31.3%) | 198 (24.7%) | 178 (22.2%) |
| Activated partial thromboplastin time | 506 (63.2%) | 250 (31.2%) | 195 (24.3%) | 174 (21.7%) |
| International normalized ratio | 505 (63.0%) | 251 (31.3%) | 198 (24.7%) | 178 (22.2%) |

*Values are n (% of the 801-patient primary analysis cohort). Declining availability in later windows may reflect discharge, clinical stability, illness severity, complications, and clinician-directed repeat testing.*
