# Supplementary material for: Dynamic perioperative thrombo-inflammatory biomarkers correlate with early functional outcomes after mechanical thrombectomy for acute ischemic stroke
Source: Front Neurosci. 2026 Oct 7;20:1922922. doi: 10.3389/fnins.2026.1922922 (PMC13684488; doi:10.3389/fnins.2026.1922922)
Supplement: Supplementary file 2 [file Supplementary_file_2.DOCX]

**Supplementary Table S2. Adjusted associations with failure of in-hospital early neurological improvement.**

| **Biomarker** | **N** | **Events** | **Adjusted OR (95% CI)** | **P value** |
| --- | --- | --- | --- | --- |
| Baseline NLR | 448 | 130 | 1.23 (1.04-1.44) | 0.015 |
| Peak NLR, days 0-7 | 536 | 154 | 1.20 (1.04-1.38) | 0.013 |
| Peak D-dimer, days 0-7 | 514 | 148 | 1.09 (1.03-1.16) | 0.002 |
| Baseline fibrinogen | 441 | 130 | 0.74 (0.58-0.96) | 0.022 |
| Minimum lymphocytes, days 0-7 | 536 | 154 | 0.65 (0.50-0.85) | 0.001 |

*Failure of early neurological improvement was defined as an admission-to-discharge NIHSS reduction <4 points without a discharge NIHSS score of 0-1. Each model was adjusted for age, sex, and admission NIHSS. Effects are scaled per interquartile-range increase.*
