# Supplementary material for: Dynamic perioperative thrombo-inflammatory biomarkers correlate with early functional outcomes after mechanical thrombectomy for acute ischemic stroke
Source: Front Neurosci. 2026 Oct 7;20:1922922. doi: 10.3389/fnins.2026.1922922 (PMC13684488; doi:10.3389/fnins.2026.1922922)
Supplement: Supplementary file 3 [file Supplementary_file_3.DOCX]

**Supplementary Table S3. Extended covariate-adjusted sensitivity analysis for unfavourable discharge outcome.**

| **Biomarker** | **N** | **Events** | **Adjusted OR (95% CI)** | **P value** |
| --- | --- | --- | --- | --- |
| Baseline NLR | 497 | 259 | 1.24 (0.99-1.56) | 0.064 |
| Peak NLR, days 0-7 | 502 | 259 | 1.24 (1.01-1.54) | 0.043 |
| Peak D-dimer, days 0-7 | 481 | 249 | 1.02 (0.96-1.09) | 0.443 |
| Baseline fibrinogen | 489 | 255 | 0.83 (0.63-1.10) | 0.190 |
| Minimum lymphocytes, days 0-7 | 502 | 259 | 0.78 (0.58-1.07) | 0.123 |
| Baseline PLR | 497 | 259 | 1.28 (0.99-1.66) | 0.062 |
| WBC, POD 4-7 | 316 | 168 | 1.45 (0.94-2.23) | 0.093 |
| WBC, POD 8-14 | 224 | 109 | 1.62 (1.07-2.46) | 0.022 |

*Each model was adjusted for age, sex, admission NIHSS, baseline ASPECTS, successful reperfusion, haemorrhagic transformation, baseline creatinine, and baseline alanine aminotransferase. Available-case denominators vary. Effects are scaled per interquartile-range increase.*
